# Symptom Network Analysis and Unsupervised Clustering of Oncology Patients Identifies Drivers of Symptom Burden and Patient Subgroups With Distinct Symptom Patterns

**DOI:** 10.1002/cam4.70278

**Published:** 2024-10-08

**Authors:** Brandon H. Bergsneider, Terri S. Armstrong, Yvette P. Conley, Bruce Cooper, Marilyn Hammer, Jon D. Levine, Steven Paul, Christine Miaskowski, Orieta Celiku

**Affiliations:** ^1^ Neuro‐Oncology Branch, National Cancer Institute National Institutes of Health Bethesda Maryland USA; ^2^ School of Medicine Stanford University Stanford California USA; ^3^ School of Nursing University of Pittsburgh Pittsburgh Pennsylvania USA; ^4^ School of Nursing University of California San Francisco San Francisco California USA; ^5^ Phyllis F Cantor Center for Research in Nursing and Patient Care Services Dana Farber Cancer Institute Boston Massachusetts USA; ^6^ School of Medicine University of California San Francisco San Francisco California USA

**Keywords:** Gaussian graphical models, network analysis, precision medicine, quality of life

## Abstract

**Background:**

Interindividual variability in oncology patients' symptom experiences poses significant challenges in prioritizing symptoms for targeted intervention(s). In this study, computational approaches were used to unbiasedly characterize the heterogeneity of the symptom experience of oncology patients to elucidate symptom patterns and drivers of symptom burden.

**Methods:**

Severity ratings for 32 symptoms on the Memorial Symptom Assessment Scale from 3088 oncology patients were analyzed. Gaussian Graphical Model symptom networks were constructed for the entire cohort and patient subgroups identified through unsupervised clustering of symptom co‐severity patterns. Network characteristics were analyzed and compared using permutation‐based statistical tests. Differences in demographic and clinical characteristics between subgroups were assessed using multinomial logistic regression.

**Results:**

Network analysis of the entire cohort revealed three symptom clusters: constitutional, gastrointestinal‐epithelial, and psychological. Lack of energy was identified as central to the network which suggests that it plays a pivotal role in patients' overall symptom experience. Unsupervised clustering of patients based on shared symptom co‐severity patterns identified six patient subgroups with distinct symptom patterns and demographic and clinical characteristics. The centrality of individual symptoms across the subgroup networks differed which suggests that different symptoms need to be prioritized for treatment within each subgroup. Age, treatment status, and performance status were the strongest determinants of subgroup membership.

**Conclusions:**

Computational approaches that combine unbiased stratification of patients and in‐depth modeling of symptom relationships can capture the heterogeneity in patients' symptom experiences. When validated, the core symptoms for each of the subgroups and the associated clinical determinants may inform precision‐based symptom management.

## Graphical abstract

Cancer patients' symptom experiences are complex and exhibit a large amount of interindividual variability. This clinical reality poses major challenges in the prioritization of symptom management interventions. This study is the first to use computational network analysis and unsupervised clustering approaches with a sample of 3088 cancer patients to model symptom interconnectedness, group patients based on shared symptom patterns, and identify core symptoms to target in each group.

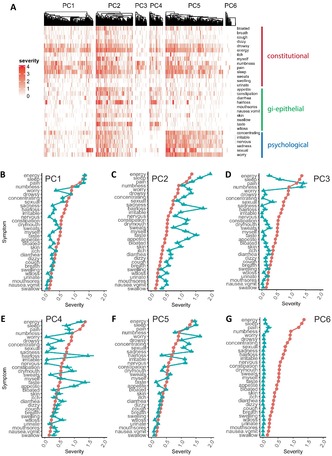

## 1 Introduction

Cancer patients experience an average of 10 (range 0–38) concurrent symptoms during treatment [1, 2] and survivors report an average of 5 (range 0–13) concurrent symptoms [3]. The number of symptoms reported in various studies is dependent on the total number of symptoms included on the assessment instrument. Equally important, this large amount of interindividual variability in symptom burden has a significant impact on patients' functional status [4, 5, 6] and quality of life [6, 7, 8]. However, the large number of symptoms; the complexity of how symptoms interact with and may synergize with one another; and the large amount of interindividual variability in patients' symptom experiences pose significant clinical challenges to the identification of core symptoms to prioritize for treatment [9]. Traditional methods that analyze the occurrence, severity and/or distress of individual symptoms do not capture the complexity and heterogeneity of cancer patients' symptom experiences.

Network analysis (NA) has emerged as a computational paradigm to model and understand the interconnectedness among symptoms [10]. Rather than considering symptoms as isolated indicators, this graph‐theory‐based approach hypothesizes that causal relationships exist between symptoms. NA ascribes roles to the symptoms based on their position in the network model; the strength of their connections to other symptoms; and the overall degree of connectivity of the network model [11]. These roles determine how impactful external stressors are in activating the collection of symptoms and how persistent the activation is when external stressors are removed. While this approach was originally developed in the context of psychopathology and has been extensively used to understand the relationships between symptoms in psychiatric disorders [11], it is more recently being used to evaluate symptoms associated with cancer and its treatments. For example, NA determined that the relationships between co‐occurring symptoms in cancer patients depended on time point in the chemotherapy cycle and type of cancer [12]. In addition, NA identified fatigue's pivotal role in driving overall symptom burden across multiple cancer types [13]; depression's centrality in the experience of patients with head and neck cancer undergoing radiotherapy [14]; and four core dimensions of the symptom experience of patients with primary brain tumors [15]. In a recent study [16], NA was used to identify core symptoms in older adults with cancer. Vomiting, fatigue, and sadness were the important symptoms in the network. In addition, the networks differed among patients with different comorbidities. These types of NA have the potential to address interindividual variability in patients' symptom experiences and provide more personalized and precise symptom management interventions.

A major limitation of NA, that is not well described, is that it is not intended to capture heterogeneity within a patient cohort. NA was pioneered in the field of psychopathology, based on the assumption that mental disorders can be conceptualized as causal systems of mutually reinforcing symptoms [11]. In other words, the collection of symptoms is the condition and NA aims to uncover its underlying mechanism(s). This assumption does not necessarily hold when applied to an evaluation of cancer symptoms because the type of cancer, treatment status, multimorbidity, social determinants of health, and other factors impact symptom dynamics and impose heterogeneity that a single symptom network cannot capture.

Although some studies have stratified patient cohorts based on cancer type, treatment status, or survivorship status [12, 13, 14, 17, 18, 19, 20, 21] and conducted NA separately, a large amount of heterogeneity remains within various subgroups that NA alone cannot capture. To address this limitation, we combine NA with a generalization of the second‐order unsupervised clustering method proposed by Henry and colleagues [22]. While traditional first‐order clustering methods, such as hierarchical clustering and latent class analysis [23], cluster patients based on direct symptom measurements, second‐order clustering groups patients based on their symptom co‐severity or co‐occurrence network dynamics. As a result, while traditional analyses group patients largely based on low and high symptom burden [24], second‐order clustering groups patients based on their unique symptom patterns [15].

Recently, we applied this combination of NA and second‐order unsupervised clustering to analyze the symptom experience of 1128 primary brain tumor patients. Of note, patient subgroups with distinct symptom patterns and clinical characteristics were identified [15]. Here, we expand the application of this novel method to symptom severity data obtained from 3088 cancer patients with heterogeneous types of cancer, to unbiasedly characterize the heterogeneity of their symptom experience and elucidate their symptom patterns and drivers of symptom burden.

## 2 Materials and Methods

### 2.1 Patients and Settings

This secondary analysis used cross‐sectional data from 3088 cancer patients who participated in one of three studies (i.e., Chemotherapy‐Induced Peripheral Neuropathy (CIPN) study [25], Symptom Clusters Study [26, 27], COVID‐19 study [28, 29]), whose details were described previously and are summarized below.

#### 2.1.1 CIPN Study

Patients (*n* = 615) were recruited from throughout the San Francisco Bay area. Patients completed questionnaires in their homes prior to an in‐person study visit. During the study visit, a research nurse obtained written informed consent, reviewed the study questionnaires for completeness, and performed the objective measures of CIPN and balance. This study was approved by the Committee on Human Research at the University of California, San Francisco (UCSF).

#### 2.1.2 Symptom Clusters Study

This longitudinal study was designed to evaluate the symptom experience of oncology patients (*n* = 1329) over 2 cycles of chemotherapy. Patients were approached in the infusion unit prior to the first or second cycle of chemotherapy. Patients who were willing to participate provided written informed consent. Data from the enrollment assessment (i.e., the week prior to the second or third cycle of chemotherapy) were used in the current analysis. This study was approved by the Committee on Human Research at UCSF and by the Institutional Review Board (IRB) at each of the study sites.

#### 2.1.3 COVID‐19 Pandemic Study

Over the course of the COVID‐19 pandemic lockdowns, oncology patients (*n* = 1142) were recruited from a registry of individuals who participated in previous symptom management studies; from electronic health record searches; and from the Dr. Susan Love Foundation for Breast Cancer Research. Patients received an email with a brief explanation of the study and a link that directed them to the study's enrollment page. This study was exempt from requiring written informed consent by the Committee on Human Research at UCSF and IRB at each of the other sites.

### 2.2 Materials

Patients completed a demographic questionnaire, the Karnofsky Performance Status (KPS) scale [30], and the Self‐Administered Comorbidity Questionnaire [31]. For the CIPN and Symptom Clusters studies, medical records were reviewed for disease and treatment information. For the COVID‐19 study, patients reported their clinical information (Table 1).

**TABLE 1:** Demographic and clinical characteristics of the all‐patient cohort and subgroups identified using unsupervised clustering.

Characteristics	Combined (*n* = 3088) *n* (%)	PC1 (*n* = 851) *n* (%)	PC2 (*n* = 639) *n* (%)	PC3 (*n* = 198) *n* (%)	PC4 (*n* = 232) *n* (%)	PC5 (*n* = 995) *n* (%)	PC6 (*n* = 171) *n* (%)
Study							
CIPN	615 (19.9)	232 (27.3)	32 (5.0)	61 (30.8)	15 (6.5)	254 (25.5)	21 (12.3)
Clusters	1329 (43.0)	279 (32.8)	551 (86.2)	29 (14.6)	198 (85.3)	222 (22.3)	50 (29.2)
COVID	1142 (37.0)	340 (40.0)	56 (8.8)	108 (54.5)	19 (8.2)	519 (52.1)	100 (58.5)
Diagnosis							
Breast	1648 (53.4)	451 (53.2)	307 (48.0)	90 (45.5)	90 (38.8)	627 (63.0)	83 (48.5)
GI	451 (14.6)	99 (11.7)	158 (24.7)	21 (10.6)	75 (32.3)	66 (6.6)	32 (18.7)
Gyn	255 (8.3)	64 (7.5)	93 (14.6)	6 (3.0)	22 (9.5)	63 (6.3)	7 (4.1)
Lung	179 (5.8)	47 (5.5)	54 (8.5)	7 (3.5)	30 (12.9)	35 (3.5)	6 (3.5)
Other/multiple	533 (17.3)	185 (21.8)	26 (4.1)	68 (34.3)	15 (6.5)	198 (19.9)	41 (24.0)
NA	20 (0.6)	5 (0.2)	1 (0.2)	6 (3.0)	0 (0)	6 (0.6)	2 (1.2)
Age							
Mean (SD)	59.7 (12.0)	62.2 (11.4)	56.5 (11.6)	65.0 (10.3)	60.5 (12.3)	57.6 (12.1)	63.6 (10.6)
Median (range)	60.9 (19.9–95.5)	63.6 (20.5–95.5)	57.1 (19.9–90.7)	65.8 (32.3–87.5)	62.0 (21.1–87.1)	58.8 (22.2–89.6)	65.6 (28.8–88.3)
Sex							
Female	2495 (80.8)	676 (79.4)	536 (83.9)	137 (69.2)	161 (69.4)	860 (86.4)	125 (73.1)
Male	570 (18.5)	170 (20.0)	100 (15.6)	59 (29.8)	71 (30.6)	126 (12.7)	44 (25.7)
NA	21 (0.7)	5 (0.6)	3 (0.5)	2 (1.0)	0 (0)	9 (0.9)	2 (1.2)
Ethnicity							
White	2373 (76.9)	691 (81.2)	426 (66.7)	161 (81.3)	140 (60.3)	834 (83.8)	121 (70.8)
Asian or Pacific Islander	248 (8.0)	58 (6.8)	74 (11.6)	14 (7.1)	42 (18.1)	44 (4.4)	16 (9.4)
Black	161 (5.2)	31 (3.6)	47 (7.4)	11 (5.6)	21 (9.1)	35 (3.5)	16 (9.4)
Hispanic, mixed, or other	286 (9.3)	64 (7.5)	88 (13.8)	12 (6.1)	24 (10.3)	81 (8.1)	17 (9.9)
NA	18 (0.6)	7 (0.8)	4 (0.6)	0 (0)	5 (2.2)	1 (0.1)	1 (0.6)
Annual income							
> $100,000	1265 (41.0)	344 (40.4)	242 (37.9)	99 (50.0)	77 (33.2)	436 (43.8)	67 (39.2)
< $100,000	1425 (46.1)	389 (45.7)	344 (53.8)	73 (36.9)	118 (50.9)	427 (42.9)	74 (43.3)
NA	396 (12.8)	118 (13.9)	53 (8.3)	26 (13.1)	37 (15.9)	132 (13.3)	30 (17.5)
KPS score							
Mean (SD)	85.3 (12.4)	86.1 (11.0)	77.2 (12.7)	90.1 (10.6)	83.7 (12.7)	87.2 (11.3)	95.6 (8.2)
Median (range)	90 (30–100)	90 (30–100)	80 (40–100)	90 (40–100)	90 (50–100)	90 (40–100)	100 (60–100)
SCQ score							
Mean (SD)	4.6 (3.5)	4.5 (3.1)	5.8 (3.6)	3.8 (3.2)	5.2 (3.5)	4.4 (3.5)	2.8 (2.9)
Median (range)	4 (0–27)	4 (0–17)	5 (0–21)	3 (0–13)	5 (0–18)	4 (0–27)	2 (0–14)
Years from diagnosis							
Mean (SD)	4.9 (6.5)	5.7 (7.0)	2.4 (4.6)	7.5 (8.0)	2.4 (4.0)	5.6 (6.2)	7.2 (8.9)
Median (range)	2.2 (0.04–55.6)	3.1 (0.1–39.6)	0.4 (0.04–44.4)	4.5 (0.1–46.0)	0.5 (0.05–23.6)	3.4 (0.04–34.9)	3.9 (0.1–55.6)
# Prior treatments							
Mean (SD)	2.3 (1.5)	2.6 (1.4)	1.7 (1.5)	2.7 (1.3)	1.5 (1.5)	2.6 (1.3)	2.0 (1.3)
Median (range)	2 (0–7)	3 (0–7)	1 (0–7)	3 (0–6)	1 (0–6)	3 (0–6)	2 (0–5)
Currently on treatment							
Yes	2004 (64.9)	519 (61.0)	586 (91.7)	83 (41.9)	208 (89.7)	526 (52.9)	82 (48.0)
No	1067 (34.6)	332 (39.0)	45 (7.0)	115 (58.1)	19 (8.2)	467 (46.9)	89 (52.0)
NA	15 (0.5)	0 (0)	8 (1.3)	0 (0)	5 (2.2)	2 (0.2)	0 (0)
Metastatic sites							
Yes	1551 (50.2)	446 (52.4)	389 (60.9)	82 (41.4)	159 (68.5)	416 (41.8)	59 (34.5)
No	1479 (47.9)	392 (46.1)	248 (38.8)	110 (55.6)	73 (31.5)	550 (55.3)	106 (62.0)
NA	56 (1.8)	13 (1.5)	2 (0.3)	6 (3.0)	0 (0)	29 (2.9)	6 (3.5)
Total QOL score							
Mean (SD)	6.0 (1.5)	6.3 (1.2)	5.2 (1.4)	7.2 (1.0)	6.5 (1.3)	5.5 (1.4)	7.6 (1.2)
Median (range)	6.1 (0.8–10)	6.5 (1.6–9.2)	5.2 (1.4–8.8)	7.3 (3.9–9.1)	6.6 (2.6–9.5)	5.7 (0.8–9.2)	7.8 (2.7–10)
Physical‐well‐being QOL score							
Mean (SD)	7.2 (1.8)	7.5 (1.5)	5.8 (1.8)	8.6 (1.2)	7.3 (1.7)	7.2 (1.6)	9.1 (1.1)
Median (range)	7.4 (0.3–10)	7.6 (2.3–10)	5.9 (0.3–10)	8.8 (3.3–10)	7.5 (1.1–10)	7.4 (0.6–10)	9.5 (3.0–10)
Psychological well‐being QOL score							
Mean (SD)	5.6 (1.8)	6.1 (1.6)	4.8 (1.7)	7.1 (1.3)	6.5 (1.7)	5.0 (1.7)	7.3 (1.5)
Median (range)	5.7 (0.3–10)	6.2 (0.7–9.6)	4.9 (0.5–9.1)	7.3 (3.8–9.9)	6.6 (1.7–9.9)	5.0 (0.3–9.2)	7.6 (2.2–10)
Social well‐being QOL score							
Mean (SD)	6.2 (2.2)	6.6 (1.9)	5.1 (2.0)	7.7 (1.7)	6.6 (1.9)	5.8 (2.2)	8.1 (1.6)
Median (range)	6.4 (0–10)	6.9 (0.1–10)	5.1 (0.3–9.6)	8.1 (0.6–10)	6.8 (1.1–9.8)	6.0 (0–10)	8.4 (2.4–10)
Spiritual well‐being QOL score							
Mean (SD)	5.2 (2.1)	5.1 (2.0)	5.5 (2.1)	5.4 (1.9)	5.7 (2.1)	4.8 (2.0)	5.9 (2.0)
Median (range)	5.1 (0–10)	5.0 (0–10)	5.4 (0.1–10)	5.3 (1.4–10)	5.7 (0.3–10)	4.7 (0–10)	5.9 (0.4–10)

Abbreviations: CIPN, chemotherapy‐induced peripheral neuropathy; KPS, Karnofsky Performance Status; QOL, quality of life; SCQ, Self‐administered Comorbidity Questionnaire; SD, standard deviation.

Patients completed the Memorial Symptom Assessment Scale (MSAS) [32] that evaluated the occurrence, frequency, severity, and distress of 32 symptoms experienced in the past week. Severity scores that ranged from 0 to 4 (i.e., 0 = did not report the symptom, 1 = slight, 2 = moderate, 3 = severe, 4 = very severe) were used in the current analyses. The MSAS has well‐established validity and reliability [33, 34].

In addition, they completed the Multidimensional Quality of Life Scale‐Patient Version (MQOLS‐PV) that assesses four domains of QOL (i.e., physical, psychological, social, and spiritual well‐being) and overall QOL. Subscale and total scores range from 0 to 10 with higher scores indicating better QOL [35]. The MQOLS‐PV has well‐established validity and reliability [36, 37, 38, 39].

### 2.3 Network Analysis

MSAS symptom severity data were used to construct Gaussian Graphical Model (GGM) networks. In a GGM symptom network, the nodes of the network (circles) represent symptoms; the edges (lines) represent the existence of conditionally dependent relationships between the severity of the symptoms; and the weights of the edges (line thickness) represent their degree of association after controlling for the contribution of the remaining symptoms [40, 41].

The optimal number of symptoms to include in networks was determined using unique variable analysis (UVA), which assesses the topological overlap of variables to identify potentially redundant pairs. Then, these redundant pairs were consolidated into a single underlying variable using the maximum likelihood with robust standard errors estimator [42].

Following redundant variable consolidation, GGMs were constructed [43, 44]. All variables were assumed to be continuous and were determined to be non‐normally distributed using the Shapiro–Wilk normality test (Figure S1B). As recommended, nonparanormal transformation was applied prior to GGM construction [41, 45]. GGMs were regularized using the Graphical Least Absolute Shrinkage and Selection Operator based on Extended Bayesian Information Criterion. This step removes potentially spurious edges from network models and leads to more accurate network architectures [40, 41, 46].

Symptom clusters in each GGM were identified using the walk trap algorithm [47]. Symptom importance in each GGM was assessed by calculating network centrality measures (i.e., strength, betweenness, closeness). Strength is a measure of how well a node (symptom) is directly connected to other nodes (symptoms). Betweenness measures how important a node is in connecting other nodes (i.e., how often a symptom serves as the common factor between other symptoms). Closeness measures how well a node (symptom) is indirectly connected to other nodes (symptoms) [40]. These centrality measures suggest which symptoms play a key role in activating or exacerbating other symptoms and contribute the most to overall symptom burden. In addition, bridge centrality measures were calculated [48]. Bridge centrality measures are calculated between nodes in different clusters. They provide insights into which symptoms are responsible for the connections between different symptom clusters [49]. Network accuracy and stability were assessed using permutation‐based statistical tests [40, 50].

### 2.4 Unsupervised Patient Clustering

Patients were divided into subgroups using unsupervised clustering on co‐severity of symptoms. This type of clustering generalizes the concordance network clustering approach described by Henry and colleagues [22]. It uses continuous symptom co‐severity data to identify communities of patients based on shared co‐severity patterns. Briefly, for each patient with reported symptom severity vector *X*, it calculates a concordance matrix *XX* ^*T*^ */100* that represents the co‐severity of symptoms for that patient. For each pair of patients, the similarity between their concordance matrices is then calculated using the Adjusted Rand Index (ARI) [51] between the two matrices. This generates a similarity matrix which is then passed into the walktrap community detection algorithm from the R‐package igraph [52] (using default parameters) to identify patient communities. Additional details on the NA steps and unsupervised clustering were described previously [15].

### 2.5 Demographic and Clinical Characteristic Comparisons

Multinomial logistic regression was used to assess demographic and clinical characteristics of subgroup membership. Patient community 1 (PC1) was used as the reference group because it was the most demographically similar to the entire patient cohort (Table 1) Models were constructed with cancer diagnosis, age, sex, current treatment status, ethnicity, income, and KPS scores as covariates. Odds‐ratios and *p* values with Bonferroni correction were calculated to assess the individual covariates' impact on subgroup membership. The R package nnet was used for these analyses [53].

## 3 Results

### 3.1 Patient Sample

Of the 3088 cancer patients (Table 1), most were female (80.8%), non‐Hispanic White (76.9%), and currently on treatment (64.9%). Breast cancer was the most common diagnosis (53.4%), followed by gastrointestinal (14.6%), gynecological (8.3%), and lung (5.3%) cancer. The mean and median ages of patients across all studies were 59.7 and 60.9 years, respectively. Mean and median KPS scores were 85.3 and 90, respectively.

### 3.2 Symptom Prevalence and Severity and QOL Scores

**FIGURE 1:**
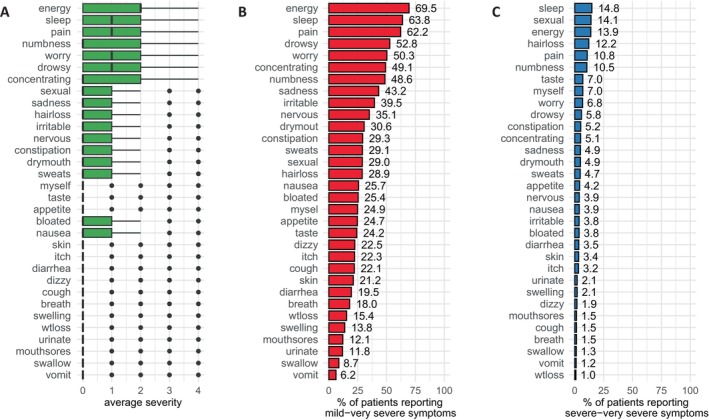
Severity of the 32 MSAS symptoms across all 3088 cancer patients and survivors: (A) Symptom severity distributions. (B) Percentage of patients reporting mild‐very severe symptoms (severity ≥ 1) and (C) severe‐very severe symptoms (severity ≥ 3).

A total of 3019 patients (97.8%) reported having at least one symptom and 1687 patients (54.6%) reported having at least one symptom that was severe or very severe (severity ≥ 3). In the sample, 23.3% reported between 1 and 5 concurrent symptoms, 34.1% reported between 6 and 10, 41.9% reported between 10 and 20, and 6.5% reported over 20 concurrent symptoms. Across all of the patients, lack of energy was the most severe symptom, followed by difficulty sleeping, pain, numbness/tingling in hands and feet, worrying, feeling drowsy, difficulty concentrating, and problems with sexual interest or activity (Figure 1). Potential redundancy between the 32 symptoms was assessed (i.e., whether any pair of symptoms likely measured the same underlying construct) by evaluating their interdependence overlap, conceptual redundancy, and outlying occurrence frequency effects. The symptom pair of nausea and vomiting met the criteria for redundancy (Figure S1A, and Figure 1) and was subsequently consolidated into a single latent variable, whose severity was computed as a mixture of the severity of the two individual symptoms. Therefore, in the NA, 31 symptoms were analyzed (30 original and 1 consolidated pair).

Across the entire cohort, the mean total QOL score was 6.0 (median = 6.1, range = 0.8–10). Mean subscale scores were as follows: physical well‐being of 7.2 (median = 7.4, range = 0.3–10), psychological well‐being of 5.6 (median = 5.7, range = 0.3–10), social well‐being of 6.2 (median = 6.4, range = 0–10), and spiritual well‐being of 5.2 (median = 5.1, range = 0–10; Table 1).

### 3.3 Symptom Clusters

**FIGURE 2:**
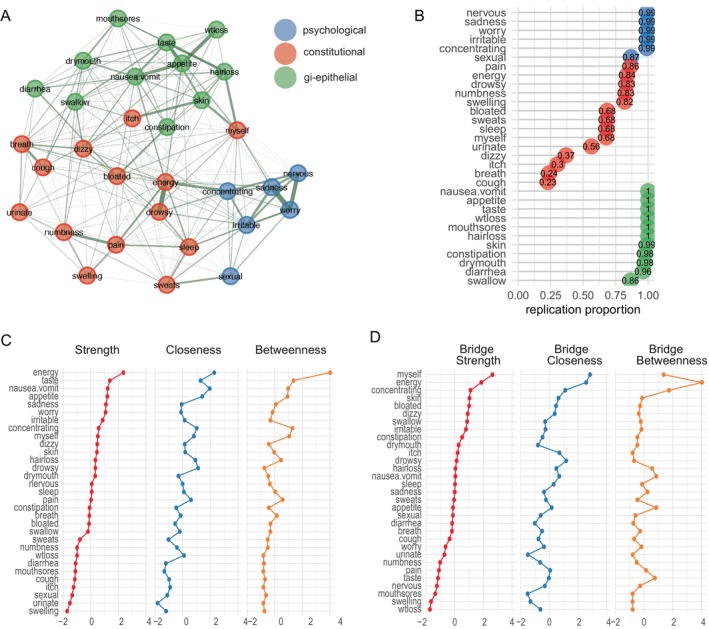
In the network for all patients and survivors, symptoms cluster into three groups: (A) A regularized Gaussian Graphical Model (GGM) was generated from the symptom severity data from all of the patients. Nodes represent symptoms. Edges represent the partial correlation between the symptom severities. Edge thickness is proportional to the strength of the correlation. Lack of edges between symptoms indicates that the regularization procedure deemed their correlations too weak to represent. All correlations were positive. Three symptom clusters were identified: Constitutional (red), gastrointestinal‐epithelial (green), and psychological (blue). (B) Symptom stability was assessed by reconstructing the network for random permutations of the dataset and calculating the proportion of times each symptom occurred in the symptom cluster shown in part A. (C) *Z*‐scored strength, closeness, and betweenness centrality for all symptoms in the network, ordered from highest to lowest according to strength centrality. (D) *Z*‐scored bridge strength, closeness, and betweenness for each symptom in the network, ordered from highest to lowest according to bridge strength centrality.

A regularized GGM network model was constructed using the 31‐symptom severity scores for the entire sample (Figure 2A). Permutation‐based statistical tests verified the accuracy and stability of the network edge weights. The symptom organization in the network was assessed for the presence of physical and psychological clusters or dimensions that explained the symptom experience. Three distinct symptom clusters were identified and labeled as constitutional, gastrointestinal‐epithelial, and psychological (Figure 2A).

Permutation‐based testing was used to evaluate the stability of the symptom clusters by repeating the symptom cluster discovery procedure for random resampling of the original dataset and calculating how often each symptom occurred in the same cluster. Exactly three clusters were identified in 74.1% of the 10,000 randomly sampled permutations. The gastrointestinal‐epithelial and psychological clusters were highly stable, as each individual symptom was found in the same cluster in over 85% of permutations (Figure 2B). The entire gastrointestinal‐epithelial and psychological clusters were replicated exactly in 87% and 86% of permutations, respectively. In contrast, the constitutional symptom cluster was not stable, as many individual symptoms in this cluster had low stability (Figure 2B) and the entire cluster was replicated exactly in only 8.6% of permutations. These results suggest that the constitutional symptom cluster serves as a “catch‐all” for generalized symptoms of cancer that do not group into the more well‐defined gastrointestinal‐epithelial or psychological symptom clusters.

### 3.4 Symptom Centralities

The high degree of connectivity in the network (i.e., 465 total connections with nonzero weights and 115 of these connections were deemed significant by permutation‐based testing at 95% confidence) suggests that activation of the symptom experience can be rapid and sustainable. To determine which symptoms are core to this activation and may have the most impact on deactivation of overall symptom burden when targeted, we calculated the symptom centrality measures of strength, closeness, and betweenness for each symptom and verified their stability using permutation‐based statistical tests. Of note, lack of energy had the highest strength, closeness, and betweenness centrality scores of all symptoms in the network (Figure 2C). This finding suggests that lack of energy substantially contributes to the severity of other symptoms and may drive many of the co‐severity relationships in the network. Other symptoms that had high values across all centrality measures (*z*‐score > 0 in all three measures) included: change in the way food tastes, nausea/vomiting, lack of appetite, difficulty concentrating, feeling that “I don't look like myself,” and hair loss (Figure 2C).

Next, we assessed which symptoms may provide activation links between the three symptom clusters and may precede or exacerbate the severity of multiple symptoms, by calculating bridge centralities and verifying their stability with permutation‐based statistical tests. Lack of energy, feeling that “I don't look like myself,” and difficulty concentrating had the highest bridge strength, bridge closeness, and bridge betweenness (Figure 2D).

Analyzing the network connections of these three symptoms, the symptoms in the network most strongly connected to (and likely activated by) lack of energy include the constitutional symptoms of feeling drowsy, pain, and difficulty sleeping; the gastrointestinal‐epithelial symptoms of nausea/vomiting and lack of appetite; and the psychological symptom of difficulty concentrating. The symptoms most strongly connected to feeling that “I don't look like myself” include the constitutional symptoms of feeling bloated and swelling of arms/legs; the gastrointestinal‐epithelial symptoms of changes in skin and hair loss; and the psychological symptoms of feeling sad, worrying, and decreased sexual interest. The symptoms most strongly connected to difficulty concentrating include the constitutional symptoms of lack of energy, difficulty sleeping, sweats, and feeling drowsy; and the psychological symptoms of feeling sad, feeling nervous, and feeling irritable (Figure 2A).

### 3.5 Patient Subgroups

**FIGURE 3:**
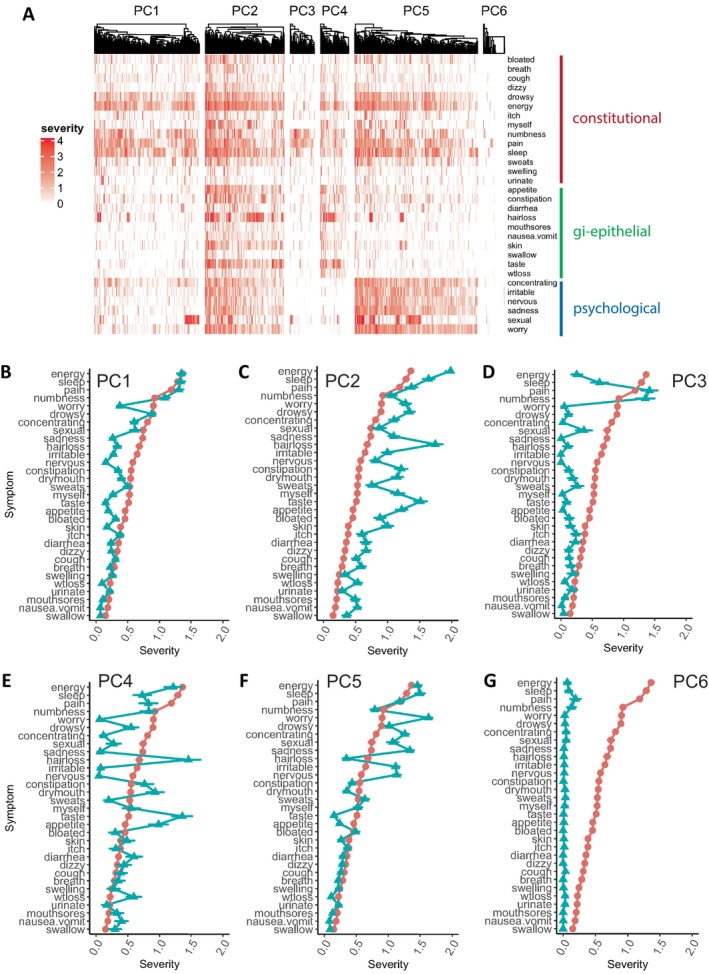
Second‐order unsupervised clustering identifies six patient subgroups with unique symptom severity patterns: (A) Heatmap of symptom severities for each patient subgroup. Symptoms are ordered based on the symptom clusters identified in Figure 2. (B–G) Average severity of each symptom in each patient subgroup (blue = average symptom severity for subgroup, red = average symptom severity for all patients). 95% confidence intervals are shown for each symptom.

The all‐patient sample analysis provides important insights into symptom clusters and dominant interaction patterns that occur across the entire sample of patients with heterogeneous types of cancer and who are at various points in their cancer trajectory. However, this type of analysis is likely to overshadow more nuanced symptom experiences of specific subgroups of patients. To assess and capture potential heterogeneity in patients' symptom experiences, we assessed the (dis)similarity of the symptom experience and sought to unbiasedly cluster patients based on shared symptom co‐severity patterns using random walk‐based unsupervised clustering. This assessment led to the identification of six patient subgroups or communities, referred to as PC1 (*n* = 851), PC2 (*n* = 639), PC3 (*n* = 198), PC4 (*n* = 232), PC5 (*n* = 995), and PC6 (*n* = 171) (Figure 3A). These clusters remained consistent across 11 separate runs of unsupervised clustering using different random walk starting positions. GGMs and network centrality measures were calculated for each subgroup (Figures S2 and S3), except for PC6. Because this subgroup had a minimal symptom burden (Figure 3A,G), the network was sparse and unstable. In addition, we compared the demographic and clinical characteristics of the subgroups (Table 1) and performed multinomial logistic regression to determine the characteristics that most strongly predicted subgroup membership (Table 2).

**TABLE 2:** Multinomial logistic regression identifies age, treatment status, and Karnofsky Performance Status (KPS) score as the most significant determinants of patient subgroup membership: Multinomial logistic regression was performed using community 1 as the reference group and comparing the variables of diagnosis, age, sex, treatment status, ethnicity, income, and KPS score.

Characteristics		PC1 (*n* = 851)		PC2 (*n* = 639)		PC3 (*n* = 198)		PC4 (*n* = 232)		PC5 (*n* = 995)		PC6 (*n* = 171)	
		OR	*p*	OR	*p*	OR	*p*	OR	*p*	OR	*p*	OR	*p*
Diagnosis													
Breast		1	—	1	—	1	—	1	—	1	—	1	—
GI		1	—	1.93	0.067	1.26	1.0	2.32	0.127	0.75	1.0	2.79	0.126
Gyn		1	—	1.56	1.0	0.77	1.0	1.62	1.0	0.80	1.0	0.97	1.0
Lung		1	—	1.51	1.0	0.57	1.0	2.34	0.589	1.04	1.0	0.74	1.0
Other/multiple		1	—	0.52	0.804	1.67	1.0	0.63	1.0	0.95	1.0	1.34	1.0
Age		1	—	**0.98**	**0.001**	1.02	1.0	1.0	1.0	**0.96**	<**0.001**	1.01	1.0
Sex													
Female	1	—	1	—	1	—	1	—	1	—	1	—	1
Male	1	—	0.72	1.0	1.22	1.0	1.28	1.0	0.72	1.0	1.01	1.0	1
Currently on treatment													
No	1	—	1	—	1	—	1	—	1	—	1	—	1
Yes	1	—	**4.18**	**< 0.001**	0.64	1.0	**3.33**	**< 0.001**	**0.64**	**0.014**	0.81	1.0	1
Ethnicity													
White	1	—	1	—	1	—	1	—	1	—	1	—	1
Asian or Pacific Islander	1	—	1.17	1.0	1.15	1.0	**2.93**	**0.002**	0.48	0.097	1.48	1.0	1
Black	1	—	1.51	1.0	2.68	0.693	2.44	0.783	0.998	1.0	3.71	0.08	1
Hispanic, mixed, or other	1	—	1.54	1.0	1.12	1.0	1.66	1.0	0.82	1.0	2.07	1.0	1
Income													
> $100,000	1	—	1	—	1	—	1	—	1	—	1	—	1
< $100,000	1	—	1.09	1.0	0.63	0.631	1.32	1.0	0.97	1.0	1.15	1.0	1
KPS score		1	—	**0.96**	**< 0.001**	**1.03**	**0.026**	1.0	1.0	1.01	1.0	**1.14**	**< 0.001**

*Note:* Bonferroni multiple comparison correction was performed and postcorrection significant odds ratios (OR) and *p* values (*p* < 0.05) are in bold. Compared to a null model (i.e., an intercept‐only model with no predictors), this model had a lower AIC (7168 vs. 8065), a higher Cragg & Uhler's pseudo *R*‐squared (0.342 vs. 0), and a log likelihood ratio test chi‐squared value of 1017 (*p* < 0.00001).

Because PC1 was demographically and clinically most representative of the entire cohort (Table 1), it was used as the reference group in multinomial logistic regression (Table 2). However, unlike in the network for the entire cohort, only a handful of symptoms (i.e., lack of energy, pain, numbness and tingling, difficulty sleeping, and drowsiness) were disproportionally represented in PC1 (Figure 3B). This subgroup appears to represent a cohort of cancer patients who are most affected by common constitutional symptoms of cancer but do not experience the gastrointestinal‐epithelial and psychological symptom clusters, or the more unique symptom patterns of some of the other subgroups. The symptoms with the highest centrality in PC1 (i.e., the most important to target in this subgroup) include lack of energy, pain, numbness and tingling, and lack of appetite (Figure S3A).

Multinomial logistic regression revealed that age, treatment status, and KPS score were the strongest determinants of PC2 membership (Table 2). PC2 was the youngest of the subgroups, had the most patients currently on treatment (92%), and had the lowest KPS scores. In addition, these patients had the lowest total, physical, psychological, and social QOL scores (Table 1). Patients in this subgroup had a very high symptom burden across all symptoms (Figure 3A,B) and their symptoms clustered into three groups: (1) constitutional, (2) psychological/changes in daily function, and (3) epithelial/changes in appearance (Figure S2B). Analyzing the centrality measurements, particularly important symptoms included: pain, lack of energy, multiple psychological symptoms (i.e., feeling worried, feeling irritable, difficulty concentrating), and lack of appetite (Figure S3B).

High KPS score was the strongest determinant of PC3 membership (Table 2). In addition, this group tended to be further out from their initial cancer diagnosis and less likely to be currently on treatment (42%) (Table 1). On average, these patients had low symptom severity scores except for numbness and pain (Figure 3D). The low symptom severity scores for this subgroup led to a sparse network (Figure S2C) which suggests that activation of individual symptoms is unlikely to lead to activation or exacerbation of other symptoms and that targeting of any severe individual symptoms is similarly effective. PC3 may represent patients who have a good prognosis or are recovered but continue to experience significant neuropathy.

Treatment status and ethnicity were the strongest determinants of PC4 membership (Table 2), as this group was more likely to be on treatment (90%) and was disproportionately Asian/Pacific Islander (18.1% compared to 8.0% across the entire cohort) (Table 1). This group had a high burden of treatment‐related symptoms such as hair loss, dry mouth, constipation, change in the way food tastes, and lack of appetite. However, compared to the mean severity scores across the entire sample, these patients had a much lower severity of psychological symptoms, less pain, less difficulty sleeping, and less drowsiness (Figure 3E). Their total, physical, psychological, social, and spiritual QOL scores were higher than average, despite having lower than average KPS scores and being most likely to have metastatic sites among all the groups (Table 1). The symptoms with the highest centralities in PC4 included lack of energy, dizziness, lack of appetite, and change in the way food tastes (Figure S3D).

Younger age and treatment status were the strongest determinants of PC5 membership (Table 2). This group was less likely to be currently on treatment (53%) and was mostly composed of female (87%) breast cancer patients (63%) (Table 1). Patients in PC5 experienced a disproportionately high burden of psychological symptoms compared to the overall sample (Figure 3F). They had lower than average total, psychological, social, and spiritual QOL scores, but average physical QOL and higher than average KPS scores (Table 1). This group may represent younger patients who may respond well to treatment but for whom the psychological burden of cancer is especially difficult. The symptoms with the highest centralities in PC5 included lack of energy, feeling sad, and feeling that “I don't look like myself” (Figure S3E).

The strongest determinant of PC6 membership was high KPS scores (Table 2), as patients in PC6 had the highest KPS scores out of all the subgroups (Table 1). This group experienced very low symptom burden across all symptoms (Figure 3G), was further out from diagnosis than average, was less likely to be currently on treatment (48%), was least likely to have metastatic sites, and had the highest QOL scores across all categories. A network could not be constructed for PC6 due to the sparsity of the co‐severity relationships.

## 4 Discussion

Combining network analysis with unbiased stratification of patients' experiences can capture complex symptom relationships across large patient samples and characterize the heterogeneity within a patient population. Our study of the symptom experience of 3088 cancer patients identified three symptom clusters at the overall sample level, namely: constitutional, gastrointestinal‐epithelial, and psychological. These clusters are consistent with previous studies that used exploratory factor analysis (EFA) to identify symptom clusters in oncology patients [1, 54]. However, EFA groups symptoms into clusters without detailing the relationships between and among individual symptoms. In contrast, NA adds details on symptom relationships within and across clusters. The consistency of NA with EFA and the added information provided by NA argue that NA may replace EFA as the standard analytic approach for symptom cluster research [10]. However, given that the findings from this study warrant replication, this suggestion requires empiric testing.

Of particular clinical relevance is the ability of NA to prioritize symptoms for management based on how central to the experience they are predicted to be. Of all of the symptoms, lack of energy had the highest centrality measures. This finding suggests that it plays a crucial role in driving overall symptom burden across the entire sample and may be the most important symptom to target clinically. This finding is consistent with other studies that found fatigue to be a central symptom in networks across various cancer types [13, 14, 15, 17, 55]. Bridge centrality analysis revealed that lack of energy is particularly important in bridging the constitutional symptoms of feeling drowsy, pain, and difficulty sleeping; the gastrointestinal‐epithelial symptoms of nausea/vomiting and lack of appetite; and the psychological symptom of difficulty concentrating. Although causal relations cannot be inferred from this type of NA, these findings suggest that the management of lack of energy, using exercise or pharmacological interventions [56], may decrease its severity and connections between other symptoms. Other symptoms identified as having high centrality or bridge centrality, and thus strong candidates for clinical prioritization, included change in the way food tastes, nausea/vomiting, lack of appetite, difficulty concentrating, feeling that “I don't look like myself,” and hair loss.

While these findings may assist with prioritization of overall symptom management interventions, variability existed among patients in their symptom experiences. While most studies that use NA fail to capture patient heterogeneity, our method of combining NA with unsupervised clustering based on shared symptom experiences identified six patient subgroups with unique symptom patterns. PC1 represented a baseline cohort of cancer patients who are most affected by common constitutional symptoms of cancer and/or its treatments. PC2 represented patients with severe symptom burden across the board. PC3 represented patients primarily experiencing neuropathy. PC4 represented patients with advanced disease and treatment‐related symptoms but with a relatively high QOL. PC5 represented younger patients for whom the psychological burden of disease was especially pronounced. PC6 represented patients with minimal symptom burden. Age, treatment status, and KPS score were the strongest determinants of subgroup membership but their interplay (in one case even with ethnicity) reaffirms the complex multi‐factorial nature of patients' symptom experiences. For each patient subgroup, symptoms with the highest network centralities, that should be prioritized for management, were identified.

Several limitations warrant consideration. First, the patient sample was highly heterogeneous with respect to diagnosis and timing of assessments across the cancer care continuum which limits an evaluation of the impact of these characteristics on symptom patterns. Second, the lack of information on medications did not allow for an examination of their potential effects on the symptom networks. Third, given that 80% of the sample was female, additional research is warranted on male patients with cancer. Fourth, the cross‐sectional design of the study precluded analysis of changes in symptom networks over time and whether these changes can be predicted or prevented.

Future directions for the use of our analytic approach include analyzing data from the same patients at different times over the course of their disease to understand if and how patients move between subgroups and which demographic and clinical characteristics predict this transition. Understanding why a patient who was doing well symptomatically deteriorated over time (such as moving from PC4 to PC2) may help identify targeted interventions to prevent this transition and vice versa. Moreover, the mechanisms that underlie subgroup membership warrant further investigation. Potential future studies include pairing symptom network analysis techniques with detailed data about patient biomarkers, tumor molecular subtypes, genetic sequencing, or co‐morbid conditions. Finally, our methods have the potential to be adapted for clinical use. For example, patient‐reported outcomes measures and demographic and clinical information could be used to automatically classify new patients into one of the six subgroups described in this paper. This categorization could assist clinicians to anticipate the need for and implement tailored symptom management interventions.

## 5 Conclusion

Using a combination of NA and second‐order unsupervised clustering on 3088 cancer patients, we found that cancer symptoms were grouped into three main symptom clusters (i.e., constitutional, gastrointestinal‐epithelial, psychological). In addition, lack of energy had the highest centrality of all of the symptoms. Moreover, we identified six patient subgroups with distinct symptom patterns, demographic characteristics, and symptom centralities, that suggest that different symptoms should be prioritized for treatment in each subgroup. These analyses demonstrate a framework for capturing patient heterogeneity while characterizing symptom network relationships in‐depth and overcoming limitations with current NA applications.

## Author Contributions

**Brandon H. Bergsneider:** conceptualization (equal), formal analysis (equal), methodology (equal), writing – original draft (equal), writing – review and editing (equal). **Terri S. Armstrong:** conceptualization (equal), funding acquisition (equal), writing – review and editing (equal). **Yvette P. Conley:** writing – review and editing (equal). **Bruce Cooper:** writing – review and editing (equal). **Marilyn Hammer:** writing – review and editing (equal). **Jon D. Levine:** writing – review and editing (equal). **Steven Paul:** writing – review and editing (equal). **Christine Miaskowski:** conceptualization (equal), funding acquisition (equal), investigation (equal), supervision (equal), writing – original draft (equal), writing – review and editing (equal). **Orieta Celiku:** conceptualization (equal), methodology (equal), supervision (equal), writing – original draft (equal), writing – review and editing (equal).

## Conflicts of Interest

The authors declare no conflicts of interest.

## IRB Statement

All three studies were approved by the Institutional Review Board at the University of California, San Francisco, and by each of the study sites.

## Supporting information


Data S1.

## Data Availability

Data are available upon request to Dr. Miaskowski and the completion of a data sharing agreement with the University of California, San Francisco.

## References

[1] C. S. Harris , K. Kober , B. Cooper , et al., “Symptom Clusters in Oncology Outpatients: Stability and Consistency Across a Cycle of Chemotherapy,” BMJ Supportive & Palliative Care 13 (2023): e1198–e1211, 10.1136/spcare-2022-003785. PMC10225477 36446517

[2] C. Miaskowski , S. M. Paul , C. S. Harris , et al., “Determination of Cutpoints for Symptom Burden in Oncology Patients Receiving Chemotherapy,” Journal of Pain and Symptom Management 63, no. 1 (2022): 42–51, 10.1016/j.jpainsymman.2021.07.018. 34333099 PMC10791137

[3] Z. Zhu , Y. Sun , Y. Kuang , et al., “Contemporaneous Symptom Networks of Multidimensional Symptom Experiences in Cancer Survivors: A Network Analysis,” Cancer Medicine 1 (2022): 663–673, 10.1002/cam4.4904. PMC9844664 35651298

[4] J. Shin , M. Hammer , M. E. Cooley , et al., “Common and Distinct Risk Factors That Influence More Severe and Distressing Shortness of Breath Profiles in Oncology Outpatients,” Cancer Medicine 13, no. 3 (2024): e7013, 10.1002/cam4.7013. 38400684 PMC10891479

[5] D. Sorrera , A. Block , L. Mackin , et al., “Decrements in Both Physical and Cognitive Function Are Associated With a Higher Symptom Burden in Oncology Patients,” Seminars in Oncology Nursing 39, no. 6 (2023): 151516, 10.1016/j.soncn.2023.151516. 37968207

[6] J. K. Davis , S. Mark , L. Mackin , et al., “Sleep Disturbance and Decrements in Morning Energy Contribute to a Higher Symptom Burden in Oncology Patients,” Sleep Medicine 108 (2023): 124–136, 10.1016/j.sleep.2023.06.004. 37354746

[7] A. Calvo‐Schimmel , M. J. Hammer , A. A. Wright , et al., “Worse Depression Profiles Are Associated With Higher Symptom Burden and Poorer Quality of Life in Patients With Gynecologic Cancer,” Cancer Nursing (2024): Published ahead of print, 10.1097/NCC.0000000000001296. PMC11263505 38259059

[8] F. Wright , B. A. Cooper , S. M. Paul , et al., “Distinct Profiles of Morning and Evening Fatigue Co‐Occurrence in Patients During Chemotherapy,” Nursing Research 72, no. 4 (2023): 259–271, 10.1097/NNR.0000000000000661. 37084242 PMC10330127

[9] N. Papachristou , P. Barnaghi , B. Cooper , et al., “Network Analysis of the Multidimensional Symptom Experience of Oncology,” Scientific Reports 9, no. 1 (2019): 2258, 10.1038/s41598-018-36973-1. 30783135 PMC6381090

[10] Z. Zhu , W. Xing , Y. Hu , B. Wu , and W. K. W. So , “Paradigm Shift: Moving From Symptom Clusters to Symptom Networks,” Asia‐Pacific Journal of Oncology Nursing 9, no. 1 (2022): 5–6, 10.1016/j.apjon.2021.12.001. 35528791 PMC9072174

[11] D. J. Robinaugh , R. H. A. Hoekstra , E. R. Toner , and D. Borsboom , “The Network Approach to Psychopathology: A Review of the Literature 2008–2018 and an Agenda for Future Research,” Psychological Medicine 50, no. 3 (2020): 353–366, 10.1017/S0033291719003404. 31875792 PMC7334828

[12] E. Kalantari , S. Kouchaki , C. Miaskowski , K. Kober , and P. Barnaghi , “Network Analysis to Identify Symptoms Clusters and Temporal Interconnections in Oncology Patients,” Scientific Reports 12, no. 1 (2022): 17052, 10.1038/s41598-022-21140-4. 36224203 PMC9556713

[13] B. H. de Rooij , S. Oerlemans , K. van Deun , et al., “Symptom Clusters in 1330 Survivors of 7 Cancer Types From the PROFILES Registry: A Network Analysis,” Cancer 127, no. 24 (2021): 4665–4674, 10.1002/cncr.33852. 34387856 PMC9291877

[14] Y. Lin , D. W. Bruner , S. Paul , et al., “A Network Analysis of Self‐Reported Psychoneurological Symptoms in Patients With Head and Neck Cancer Undergoing Intensity‐Modulated Radiotherapy,” Cancer 128, no. 20 (2022): 3734–3743, 10.1002/cncr.34424. 35969226 PMC9529994

[15] B. H. Bergsneider , E. Vera , O. Gal , et al., “Discovery of Clinical and Demographic Determinants of Symptom Burden in Primary Brain Tumor Patients Using Network Analysis and Unsupervised Clustering,” Neuro‐Oncology Advances 5, no. 1 (2023): vdac188, 10.1093/noajnl/vdac188. 36820236 PMC9938652

[16] Y. Kuang , F. Jing , Y. Sun , Z. Zhu , and W. Xing , “Symptom Networks in Older Adults With Cancer: A Network Analysis,” Journal of Geriatric Oncology 15, no. 3 (2024): 101718, 10.1016/j.jgo.2024.101718. 38340638

[17] S. Y. Rha and J. Lee , “Stable Symptom Clusters and Evolving Symptom Networks in Relation to Chemotherapy Cycles,” Journal of Pain and Symptom Management 61, no. 3 (2021): 544–554, 10.1016/j.jpainsymman.2020.08.008. 32828931

[18] J. G. Röttgering , T. M. C. K. Varkevisser , M. Gorter , et al., “Symptom Networks in Glioma Patients: Understanding the Multidimensionality of Symptoms and Quality of Life,” Journal of Cancer Survivorship 16 (2023): 1032–1041, 10.1007/s11764-023-01355-8. PMC11082018 36922442

[19] F. Jing , Z. Zhu , J. Qiu , et al., “Contemporaneous Symptom Networks and Correlates During Endocrine Therapy Among Breast Cancer Patients: A Network Analysis,” Front Oncologia 13 (2023): 101786, accessed July 24, 2023, 10.3389/fonc.2023.1081786. PMC10103712 37064124

[20] T. J. Hartung , E. I. Fried , A. Mehnert , A. Hinz , and S. Vehling , “Frequency and Network Analysis of Depressive Symptoms in Patients With Cancer Compared to the General Population,” Journal of Affective Disorders 256 (2019): 295–301, 10.1016/j.jad.2019.06.009. 31200167

[21] E. J. Shim , H. Ha , Y. S. Suh , et al., “Network Analyses of Associations Between Cancer‐Related Physical and Psychological Symptoms and Quality of Life in Gastric Cancer Patients,” Psycho‐Oncology 30, no. 6 (2021): 946–953, 10.1002/pon.5681. 33760355

[22] T. R. Henry , S. A. Marshall , N. E. Avis , B. J. Levine , and E. H. Ip , “Concordance Networks and Application to Clustering Cancer Symptomology,” PLoS One 13, no. 3 (2018): e0191981, 10.1371/journal.pone.0191981. 29538418 PMC5851541

[23] N. Papachristou , C. Miaskowski , P. Barnaghi , et al., “Comparing Machine Learning Clustering With Latent Class Analysis on Cancer Symptoms' Data,” 2016. In 2016 IEEE Healthcare Innovation Point‐Of‐Care Technologies Conference (HI‐POCT). pp. 162–166, 10.1109/HIC.2016.7797722.

[24] M. Tejada , C. Viele , K. M. Kober , et al., “Identification of Subgroups of Chemotherapy Patients With Distinct Sleep Disturbance Profiles and Associated Co‐Occurring Symptoms,” Sleep 42, no. 10 (2019): zsz151, 10.1093/sleep/zsz151. 31361899 PMC6783885

[25] C. Miaskowski , J. Mastick , S. M. Paul , et al., “Chemotherapy‐Induced Neuropathy in Cancer Survivors,” Journal of Pain and Symptom Management 54, no. 2 (2017): 204–218.e2, 10.1016/j.jpainsymman.2016.12.342. 28063866 PMC5496793

[26] C. Miaskowski , B. A. Cooper , M. Melisko , et al., “Disease and Treatment Characteristics Do Not Predict Symptom Occurrence Profiles in Oncology Outpatients Receiving Chemotherapy,” Cancer 120, no. 15 (2014): 2371–2378, 10.1002/cncr.28699. 24797450 PMC4108553

[27] C. Miaskowski , B. A. Cooper , B. Aouizerat , et al., “The Symptom Phenotype of Oncology Outpatients Remains Relatively Stable From Prior to Through 1 Week Following Chemotherapy,” European Journal of Cancer Care 26, no. 3 (2017): e12437, 10.1111/ecc.12437. PMC7233145 26777053

[28] C. Miaskowski , S. M. Paul , K. Snowberg , et al., “Loneliness and Symptom Burden in Oncology Patients During the COVID‐19 Pandemic,” Cancer 127, no. 17 (2021): 3246–3253, 10.1002/cncr.33603. 33905528 PMC9508796

[29] C. Miaskowski , S. M. Paul , K. Snowberg , et al., “Stress and Symptom Burden in Oncology Patients During the COVID‐19 Pandemic,” Journal of Pain and Symptom Management 60, no. 5 (2020): e25–e34, 10.1016/j.jpainsymman.2020.08.037. 32889039 PMC7462969

[30] D. Karnofsky , “Factors That Influence the Therapeutic Response in Cancer: A Comprehensive Treatise,” in Performance Scale, eds. G. T. Kenealey and M. S. Mitchell (New York: Plenum Press, 1977).

[31] O. Sangha , G. Stucki , M. H. Liang , A. H. Fossel , and J. N. Katz , “The Self‐Administered Comorbidity Questionnaire: A New Method to Assess Comorbidity for Clinical and Health Services Research,” Arthritis and Rheumatism 49, no. 2 (2003): 156–163, 10.1002/art.10993. 12687505

[32] R. K. Portenoy , H. T. Thaler , A. B. Kornblith , et al., “The Memorial Symptom Assessment Scale: An Instrument for the Evaluation of Symptom Prevalence, Characteristics and Distress,” European Journal of Cancer 30, no. 9 (1994): 1326–1336, 10.1016/0959-8049(94)90182-1. 7999421

[33] M. Chen , S. Li , G. Jin , R. Li , Z. Qi , and Y. He , “Symptom Clusters and Network Analysis of Patients With Intermediate and Advanced Liver Cancer Treated With Targeted Immunotherapy,” Supportive Care in Cancer 32, no. 9 (2024): 580, 10.1007/s00520-024-08784-w. 39115725

[34] X. Yang , J. Bai , J. Zhang , Y. Wang , H. Zhao , and X. Zhu , “Symptom Clusters and Their Impacts on the Quality of Life of Patients With Lung Cancer Receiving Immunotherapy: A Cross‐Sectional Study,” Journal of Clinical Nursing (2024): 1–16, 10.1111/jocn.17321. 38886988

[35] B. R. Ferrell , C. Wisdom , and C. Wenzl , “Quality of Life as an Outcome Variable in the Management of Cancer Pain,” Cancer 63, no. 11 (1989): 2321–2327, 10.1002/1097-0142(19890601)63:11<2321::AID-CNCR2820631142>3.0.CO;2-T. 2720579

[36] R. Pinar , “Reliability and Validity of the Turkish Version of Multidimensional Quality of Life Scale—Cancer Version 2 in Patients With Cancer,” Cancer Nursing 27, no. 3 (2004): 252–257. 15238814 10.1097/00002820-200405000-00012

[37] G. V. Padilla , B. Ferrell , M. M. Grant , and M. Rhiner , “Defining the Content Domain of Quality of Life for Cancer Patients With Pain,” Cancer Nursing 13, no. 2 (1990): 108–115. 2331691

[38] M. Grant , G. V. Padilla , B. R. Ferrell , and M. Rhiner , “Assessment of Quality of Life With a Single Instrument,” Seminars in Oncology Nursing 6, no. 4 (1990): 260–270, 10.1016/0749-2081(90)90028-4. 2274723

[39] B. R. Ferrell , K. H. Dow , and M. Grant , “Measurement of the Quality of Life in Cancer Survivors,” Quality of Life Research 4, no. 6 (1995): 523–531, 10.1007/BF00634747. 8556012

[40] S. Epskamp , D. Borsboom , and E. I. Fried , “Estimating Psychological Networks and Their Accuracy: A Tutorial Paper,” Behavior Research Methods 50, no. 1 (2018): 195–212, 10.3758/s13428-017-0862-1. 28342071 PMC5809547

[41] S. Epskamp , “A Tutorial on Regularized Partial Correlation Networks,” Psychological Methods 23, no. 4 (2018): 617–634, 10.1037/met0000167. 29595293

[42] A. P. Christensen , L. E. Garrido , and H. Golino , “Unique Variable Analysis: A Network Psychometrics Method to Detect Local Dependence,” Multivariate Behavioral Research 58 (2023): 1–18, 10.1080/00273171.2023.2194606. 37139938

[43] S. Epskamp , A. O. J. Cramer , L. J. Waldorp , V. D. Schmittmann , and D. Borsboom , “Qgraph: Network Visualizations of Relationships in Psychometric Data,” Journal of Statistical Software 48 (2012): 1–18, 10.18637/jss.v048.i04.

[44] H. Golino , “Exploratory Graph Analysis—a Framework for Estimating the Number of Dimensions in Multivariate Data Using Network Psychometrics,” 2021, https://cran.r‐project.org/web/packages/EGAnet/index.html.

[45] H. Liu , J. Lafferty , and L. Wasserman , “The Nonparanormal: Semiparametric Estimation of High Dimensional Undirected Graphs,” Journal of Machine Learning Research 10, no. 80 (2009): 2295–2328. PMC4729207 26834510

[46] S. Epskamp , G. K. J. Maris , L. J. Waldorp , and D. Borsboom , “Network Psychometrics,” arXiv (2018): 953–986, 10.48550/arXiv.1609.02818. 29111774

[47] H. F. Golino and S. Epskamp , “Exploratory Graph Analysis: A New Approach for Estimating the Number of Dimensions in Psychological Research,” PLoS One 12, no. 6 (2017): e0174035, 10.1371/journal.pone.0174035. 28594839 PMC5465941

[48] P. Jones , “Networktools: Tools for Identifying Important Nodes in Networks,” 2021 accessed March 9, 2022, https://CRAN.R‐project.org/package=networktools.

[49] P. J. Jones , R. Ma , and R. J. McNally , “Bridge Centrality: A Network Approach to Understanding Comorbidity,” Multivariate Behavioral Research 56, no. 2 (2021): 353–367, 10.1080/00273171.2019.1614898. 31179765

[50] A. P. Christensen and H. Golino , “Estimating the Stability of Psychological Dimensions via Bootstrap Exploratory Graph Analysis: A Monte Carlo Simulation and Tutorial,” Psychiatry 3, no. 3 (2021): 479–500, 10.3390/psych3030032.

[51] L. Hubert and P. Arabie , “Comparing Partitions,” Journal of Classification 2, no. 1 (1985): 193–218, 10.1007/BF01908075.

[52] G. Csardi , T. Nepusz , V. Traag , et al., “igraph: Network Analysis and Visualization in R,” accessed August 17, 2024, https://igraph.org/.

[53] B. Ripley and W. Venables , “nnet: Feed‐Forward Neural Networks and Multinomial Log‐Linear Models,” 2023 accessed July 24, 2023, https://cran.r‐project.org/web/packages/nnet/index.html.

[54] H. M. Skerman , P. M. Yates , and D. Battistutta , “Multivariate Methods to Identify Cancer‐Related Symptom Clusters,” Research in Nursing & Health 32, no. 3 (2009): 345–360, 10.1002/nur.20323. 19274688

[55] T. Cai , T. Zhou , Q. Huang , F. Wu , F. Ni , and C. Yuan , “Cancer–Related Symptoms Among Young and Middle–Aged Women Undergoing Chemotherapy for Breast Cancer: Application of Latent Class Analysis and Network Analysis,” European Journal of Oncology Nursing 63 (2023): 102287, 10.1016/j.ejon.2023.102287. 36889245

[56] D. P. Lawrence , B. Kupelnick , K. Miller , D. Devine , and J. Lau , “Evidence Report on the Occurrence, Assessment, and Treatment of Fatigue in Cancer Patients,” Journal of the National Cancer Institute. Monographs 2004, no. 32 (2004): 40–50, 10.1093/jncimonographs/lgh027. 15263040

