# Supplementary material for: Symptom Network Analysis and Unsupervised Clustering of Oncology Patients Identifies Drivers of Symptom Burden and Patient Subgroups With Distinct Symptom Patterns
Source: Cancer Med. 2024 Oct 8;13(19):e70278. doi: 10.1002/cam4.70278 (PMC11460217; doi:10.1002/cam4.70278)
Supplement: Supplementary file 1 — Data S1. [file CAM4-13-e70278-s001.pptx]

## Slide 1
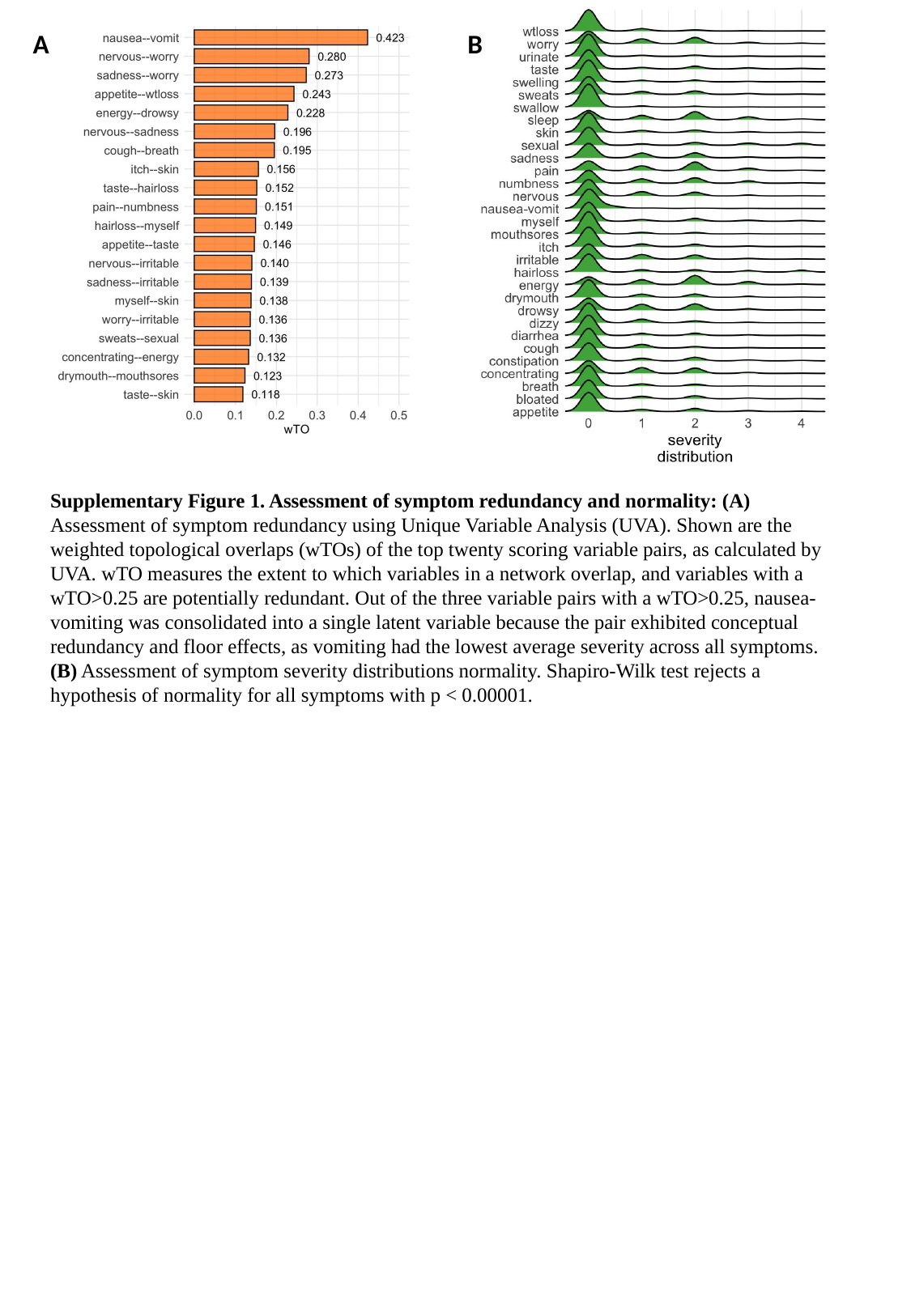

A
B
Supplementary Figure 1. Assessment of symptom redundancy and normality: (A) Assessment of symptom redundancy using Unique Variable Analysis (UVA). Shown are the weighted topological overlaps (wTOs) of the top twenty scoring variable pairs, as calculated by UVA. wTO measures the extent to which variables in a network overlap, and variables with a wTO>0.25 are potentially redundant. Out of the three variable pairs with a wTO>0.25, nausea-vomiting was consolidated into a single latent variable because the pair exhibited conceptual redundancy and floor effects, as vomiting had the lowest average severity across all symptoms. (B) Assessment of symptom severity distributions normality. Shapiro-Wilk test rejects a hypothesis of normality for all symptoms with p < 0.00001.

## Slide 2
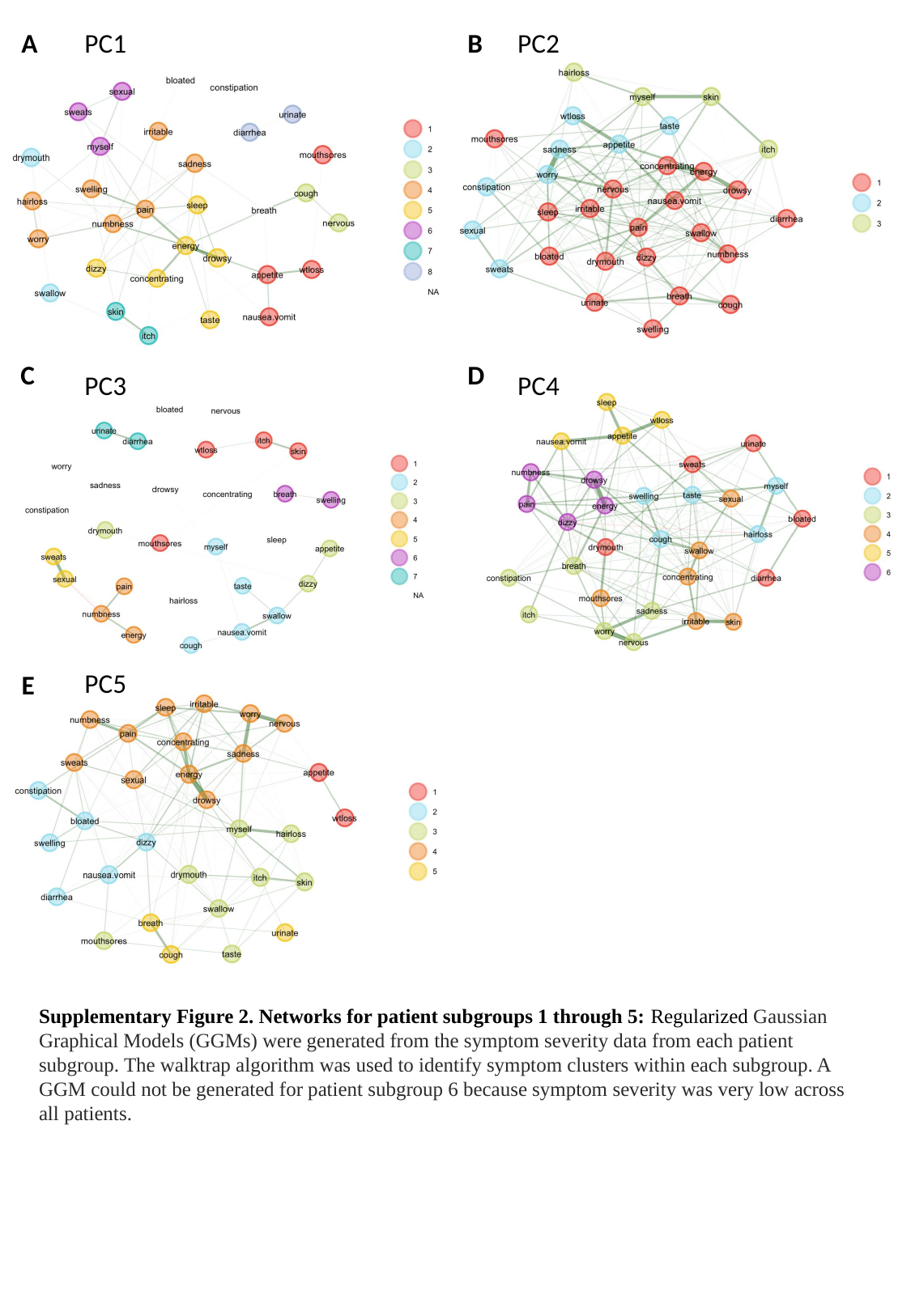

A
PC1
B
PC2
D
C
PC3
PC4
PC5
E
Supplementary Figure 2. Networks for patient subgroups 1 through 5: Regularized Gaussian Graphical Models (GGMs) were generated from the symptom severity data from each patient subgroup. The walktrap algorithm was used to identify symptom clusters within each subgroup. A GGM could not be generated for patient subgroup 6 because symptom severity was very low across all patients.

## Slide 3
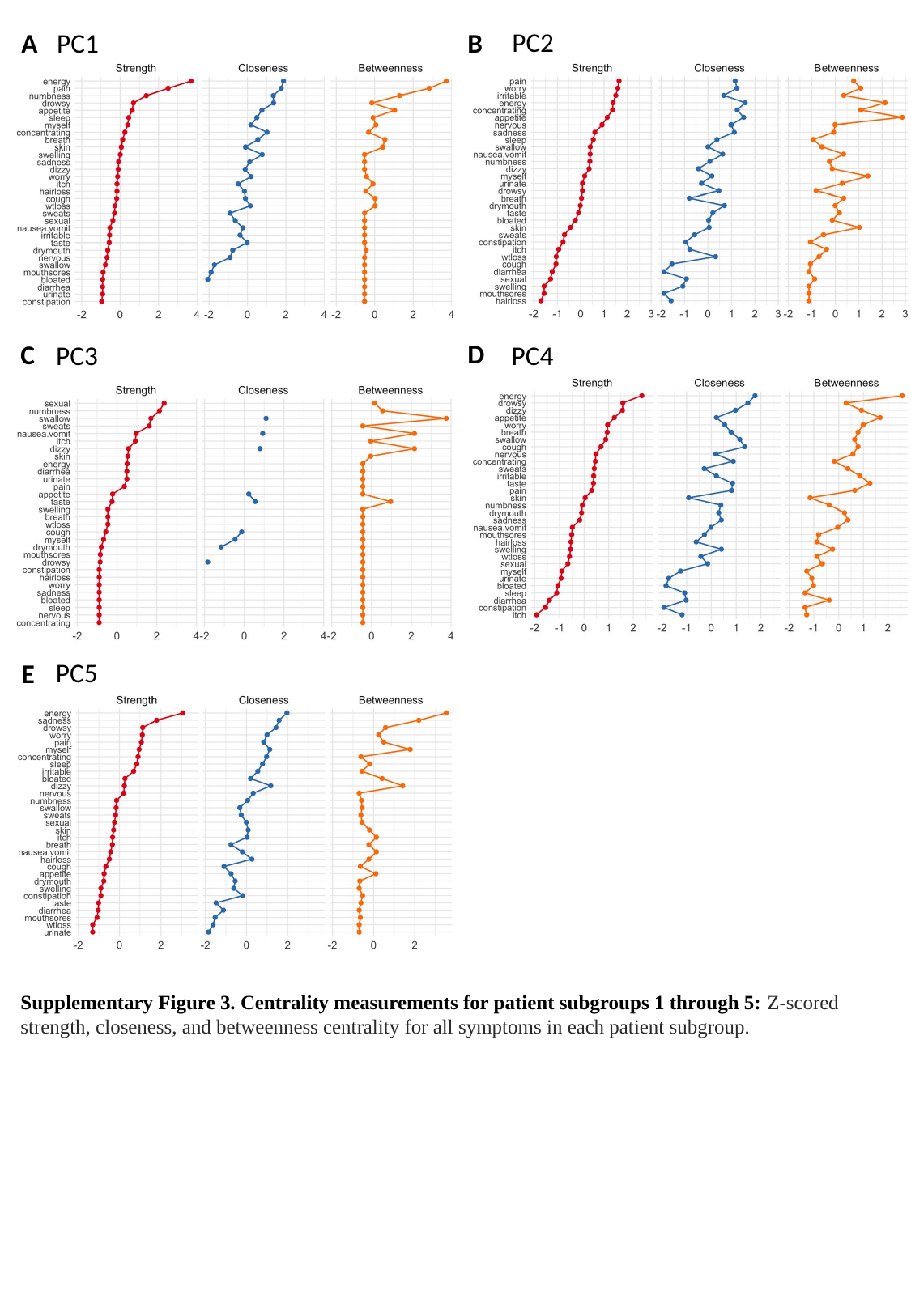

PC2
A
PC1
B
D
C
PC3
PC4
PC5
E
Supplementary Figure 3. Centrality measurements for patient subgroups 1 through 5: Z-scored strength, closeness, and betweenness centrality for all symptoms in each patient subgroup.
